# Cytomegalovirus-induced salivary gland pathology: resistance to kinase inhibitors of the upregulated host cell EGFR/ERK pathway is associated with CMV-dependent stromal overexpression of IL-6 and fibronectin

**DOI:** 10.1186/2042-4280-4-1

**Published:** 2013-01-23

**Authors:** Michael Melnick, Parish P Sedghizadeh, Krysta A Deluca, Tina Jaskoll

**Affiliations:** 1 Laboratory for Developmental Genetics, University of Southern California, 925 W 34th Street, MC-0641, Los Angeles, CA 90089-0641, USA; 2 Oral and Maxillofacial Pathology, Division of Diagnostic Sciences, University of Southern California, Los Angeles, CA, USA

**Keywords:** Cytomegalovirus, Salivary gland, Tumorigenesis, EGFR, ERK, Fibronectin, Interleukin 6

## Abstract

**Background:**

Recently we identified a relationship between human cytomegalovirus (hCMV) and human salivary gland (SG) mucoepidermoid carcinoma (MEC) in over 90% of cases; tumorigenesis in these cases uniformly correlated with active hCMV protein expression and an upregulation of the EGFR → ERK pathway. Our previously characterized, novel mouse organ culture model of mouse CMV (mCMV)-induced tumorigenesis displays a number of histologic and molecular characteristics similar to human MEC.

**Methods:**

Newborn mouse submandibular glands (SMGs) were incubated with 1 × 10^5^ PFU/ml of *lacZ*-tagged mCMV RM427+ on day 0 for 24 hours and then cultured in virus-free media for a total of 6 or 12 days with or without EGFR/ERK inhibitors and/or aciclovir. SMGs were collected for histology, immunolocalization (pERK, FN, IL-6), viral distribution, or Western blot analysis (pERK).

**Results:**

Here we report: (1) mouse SMG tumors soon exhibit an acquired resistance to EGFR/ERK pathway kinase inhibitors, alone or in combination; (2) long term tumor regression can only be sustained by concurrent inhibitor and antiviral treatment; (3) CMV-dependent, kinase inhibitor resistance is associated with overexpression of fibronectin and IL-6 proteins in abnormal stromal cells.

**Conclusions:**

Acquired resistance to kinase inhibitors is dependent upon CMV dysregulation of alternative pathways with downstream effectors common with the targeted pathway, a phenomenon with important therapeutic implications for human MEC of salivary glands.

## Background

Mucoepidermoid carcinoma (MEC) is the most common malignant tumor originating in major and minor salivary glands (SGs), accounting for about 1/3 of all SG carcinomas [1,2]. Recently, we identified a relationship between human cytomegalovirus (hCMV) and human SG MEC in over 90% of cases; tumorigenesis in these cases uniformly correlates with active hCMV protein expression and an upregulation and activation of the EGFR→ ERK pathway [3]. Concomitant with this finding, we have developed a novel mouse SG organ culture model of mouse CMV (mCMV)-induced tumorigenesis that displays a number of histologic and molecular characteristics similar to human MEC [4-6]. Specifically, mCMV-induced SG dysplasia/neoplasia is characterized by (1) mesenchymal-to-epithelial transformation (MET); (2) epithelial islands comprised of hyperplastic, dysplastic and neoplastic cells; (3) an admixing of basophilic stromal and abnormal epithelial cells; (4) migration of epithelial cells into dilated ductal lumina; (5) *de novo* re-expression of CREB-regulated transcription coactivator 1 (CRTC1) protein, a protein found in SG MECs but not in normal SG tissue [7]; and (6) an upregulation of the activated COX/AREG/EGFR/ERK signaling pathway. Further, in the short term, the mCMV-induced neoplastic phenotype can be partially rescued by inhibitors of COX (diclofenac) and EGFR (gefitinib), and fully rescued by an inhibitor of MEK1/2-mediated ERK1/2 phosphorylation (U10126), as well as by the antiviral, aciclovir.

Here we report that although EGFR/ERK pathway inhibition initially attenuates tumor progression and induces tumor regression, it is uniformly limited by an acquired drug resistance, and subsequent failure to sustain either tumor regression or stability. This drug resistance appears to be dependent upon CMV dysregulation of alternative pathways with downstream effectors common with the targeted pathway. These observations likely have important therapeutic implications for human salivary gland tumors.

## Materials and methods

### Animals

Timed pregnant inbred C57/BL6 female mice were purchased from Charles River (Wilmington, MA) [plug day = day 0 of gestation] and newborn (NB) mice were harvested as previously described [6,8]. All protocols involving mice were approved by the Institutional Animal Care and Use Committee (USC, Los Angeles, CA).

### Organ culture

Newborn (NB) SMGs were dissected and cultured for 6 (NB + 6) or 12 (NB + 12) days using a 3D organ culture system and BGJb medium (Invitrogen Corporation, Carlsbad, CA) as previously described [6]. This organ culture system maintains the morphological integrity, 3D architecture and microenvironment associations between acinar, ductal and stromal cells seen in *in vivo* SMGs. Briefly, SMG organs were cultured on small discs of Nucleopore filter (150 μm thick, with 0.8 μm pores), which in turn were placed upon a stainless steel supporting grid (~15-25 filters per grid). The grids were then placed on the inner ring of Grobstein culture dishes and 1 ml of medium was added to the well below the grid. The SMG organs develop at the air/medium interface, with the 1 × 10^5^ plaque-forming units (PFU)/ml of *lacZ*-tagged mCMV RM427+ [9] in BGJb medium being below the grid on day 0 for 24 hrs and then with virus-free medium with/without treatment for the remaining culture period. Media with or without drugs was changed daily. SMG 3D organs were not “bathed in” mCMV-infected medium as in cell and tissue culture systems, exposed to virus-infected medium for the entire culture period, nor inoculated with virus. Controls consisted of SMG organs cultured in uninfected media. SMGs were collected and processed for hematoxylin and eosin histology, immunolocalization, viral distribution or Western blot analysis. For histology, and immmunolocalization analysis, SMGs were fixed for 4 hours in Carnoy’s fixative at 4°C or overnight in 10% neutral buffered formalin at room temperature, embedded in low melting point paraplast, serially-sectioned at 8 μm and stained as previously described [6].

Recombinant virus: *lac*Z-tagged recombinant mCMV RM427+ (kindly provided by Dr. Edward Mocarski) was derived from murine CMV strain K181^+^ by insertion of a *lac*Z expression cassette driven by a human CMV *ie*1/*ie*2 gene promoter fragment [9]. To obtain a measure of mCMV infection, we assayed for β-galactosidase (*lacZ*) activity as previously described [6]. Briefly, NB + 6 and NB + 12 SMGs were fixed in 0.2% gluteraldehyde in PBS and stained for18 hrs at room temperature in standard staining solution (5 mM potassium ferricyanide, 5 mM potassium ferrocyanide, 2 mM MgCl2, 0.4% X-gal in PBS). Whole mounts were then dehydrated through graded alcohols, embedded in paraffin, serially-sectioned at 8 μm and counterstained with eosin. β-galactosidase-stained virus appears dark blue and uninfected cells appear pink.

### Immunolocalization

Cultured SMG organs were embedded in low melting point paraplast, serially-sectioned at 8 μm and immunostained as previously described [6,8] using the following commercially-available polyclonal antibodies: pERK1/2 (Thr202/Tyr204); IL-6 (Santa Cruz Biotechnology, Inc, Santa Cruz, CA) and fibronectin (Sigma-Aldrich, St. Louis, MO). Nuclei were counterstained with DAPI (Invitrogen Corporation). Negative controls were performed in parallel under identical conditions and consisted of sections incubated without primary antibodies. For each treatment group, 3–6 SMGs per day were analyzed. All images were acquired with a Zeiss Axioplan microscope equipped with a SPOT RT3 camera and processed with SPOT Advanced and Adobe Photoshop CS2 software.

### Western blot analysis

**U**ninfected (control), mCMV-infected, and GEF-treated mCMV-infected NB + 6 SMGs were collected; each independent sample consisted of 3–4 explants per group. Proteins (25-35μg) were separated by SDS-PAGE gels and transferred to a PVDF membrane, and the membranes were subjected to chemiluminescence detection (ECL) according to the manufacturer’s instructions (ThermoScientific, Rockford, IL) as previously described [6]. Antibodies: pERK1/2 (Thr202/Tyr204) and β-actin (Santa Cruz Biotechnology). Data was quantitated using the ImageJ image analysis software (NIH) and normalized to the level of β-actin expression in each sample.

### Statistical analysis

Significant differences between mCMV-infected and control SMGs, as well as between mCMV and mCMV + treatment SMGs, were determined by student t-test, with α = 0.05 and the null hypothesis of R = 1. The calculated expression ratios (Rs) were log or arcsin transformed prior to analysis.

### Interruption studies

We conducted 3 sets of interruption studies: (1) *Targeting of EGFR or ERK signaling*: To target EGFR signaling, we employed 10 μM gefitinib (GEF) (Selleck Chemicals LLC, Houston, TX), a small molecule inhibitor which blocks the binding of ATP to the intracellular TK domain of EGFR to inhibit EGFR signaling, as described previously [5]. To interrupt ERK signaling, we employed 10 μM U0126 (EMD Chemicals, Inc, Gibbstown, NJ), a potent and specific inhibitor of MEK-mediated ERK1 and ERK2 phosphorylation, as previously described [5]. These concentrations were previously shown to be the optimal, nontoxic dose that substantially precludes mCMV-induced pathology on day 6 of culture [5]. NB SMG organs were infected with 1 × 10^5^ PFU/ml mCMV for 24 hr in the presence or absence of either 10 μM GEF or 10 μM U0126 and then cultured in control medium + inhibitor for a total of 6 or 12 days. Controls consisted of SMGs cultured in control medium, 10 μM GEF-supplemented media or 10 μM U0126-supplemented media. No differences were seen between untreated, GEF-treated, and U0126-treated controls; we present untreated controls. (2) *Co-targeted inhibition of the EGF →ERK pathway*: NB SMG organs were infected with 1 × 10^5^ PFU/ml mCMV for 24 hr in the presence or absence of either 10 μM GEF or 10 μM U0126 and then in control medium + inhibitor. Beginning on day 6, we additionally added either 10 μM U1026 (GEF + D6U) or 10 μM GEF (U + D6 GEF) to the culture medium for an additional 6 days; SMGs were cultured for a total of 12 days (NB + 12). Controls consisted of untreated, GEF + D6U-treated or U + D6 GEF-treated SMGs. No differences between treatment groups were seen; untreated controls are presented. (3) *Dependency on CMV*: We used 10 μg/ml aciclovir sodium (American Pharmaceutical Partners, Inc, Schaumberg, IL), a synthetic purine nucleoside analogue which is a highly selective agent for CMV with low toxicity to the host cell [10] and previously shown to inhibit mCMV infection in mouse SMGs *in vitro*[6]; this concentration was previously shown to be the optimal, nontoxic dose [6]. NB SMG organs were infected with 1 × 10^5^ PFU/ml mCMV in control medium, 10 μM GEF-supplemented medium or 10 μM U0126-supplemented medium for 24 hr and then in control medium + inhibitor until day 6. Beginning on day 6, we added 10 μg/ml aciclovir to the medium (D6Acy; U + D6Acy; GEF + D6Acy) and cultured for an additional 6 days; SMGs were cultured for a total of 12 day (NB + 12). Controls consisted of untreated, D6Acy, U + D6Acy-treated, or GEF + D6Acy-treated media. No differences between treatment groups were seen; untreated controls are presented. For all *in vitro* studies, media was changed daily; thus new drug treatments were added daily.

## Results

The embracing paradigm of this line of investigation is to identify molecular targets critical to altering phenotypic outcome as to preclude or treat human salivary gland tumors, specifically those associated with active CMV infection. To this end, we employ an *in vitro* submandibular salivary gland (SMG) 3D organ culture strategy shown to induce cellular pathology which resembles secretory glandular neoplasia [4-6]. This SMG organ culture system maintains the three-dimensional architecture and microenvironment associations between acinar, ductal and stromal cells seen in *in vivo* glands.

**Figure 1:**
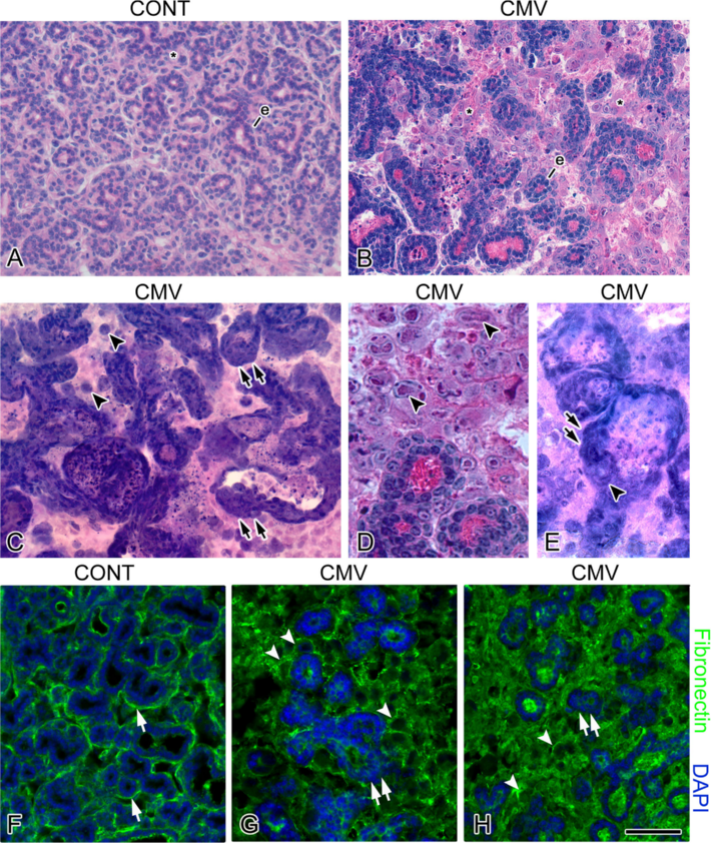
**Histologic morphology and cell-specific expression of fibronectin in control and mCMV-infected NB+12 SMGs. A**-**E**. Histologic analyses. **F**-**H**. Fibronectin (FN) immunolocalization. Control SMGs (**A**) are composed of densely packed, branched epithelial ducts and pro-acini (e) surrounded by fibromyxoid stroma. mCMV-infected SMGs (**B**-**E**) show significant viral CPE in the stroma and epithelial dysplasia. There is a notable decrease in branched epithelia (e), hyperplastic and pseudostratified epithelial ducts with dilated lumina, abnormal multilayered epithelial islands lacking a distinct BMZ and reduced cell-to-cell junctions, and there appears to be an admixing of the basophilic stromal and pro-acinar epithelial cells (double black arrows). Abnormal epithelial islands are embedded in a hypercellular stroma (*) composed of 2 distinct cell types: small eosinophilic cells and large basophilic, pleiomorphic cells. Kidney-shaped nuclei pathognomonic of mCMV infection (black arrowheads) are seen in abnormal stromal and ductal cells. **F**-**H**. FN immunolocalization. Control SMGs (**F**) show a distinct and well-demarcated pattern of FN immunoreactivity at the BMZ (white arrow). In mCMV-infected SMGs (**G**-**H**), there is a marked shift in FN immunostaining: immunodetectable FN surrounds individual metaplastic stromal cells (white arrowheads) and is nearly absent from epithelial BMZ (double white arrows). FN immunoreactivity is also seen filling ductal lumina. Bar: **A**-**D**, **F**-**H**- 50 μm; **E**- 60 μm.

**Figure 2:**
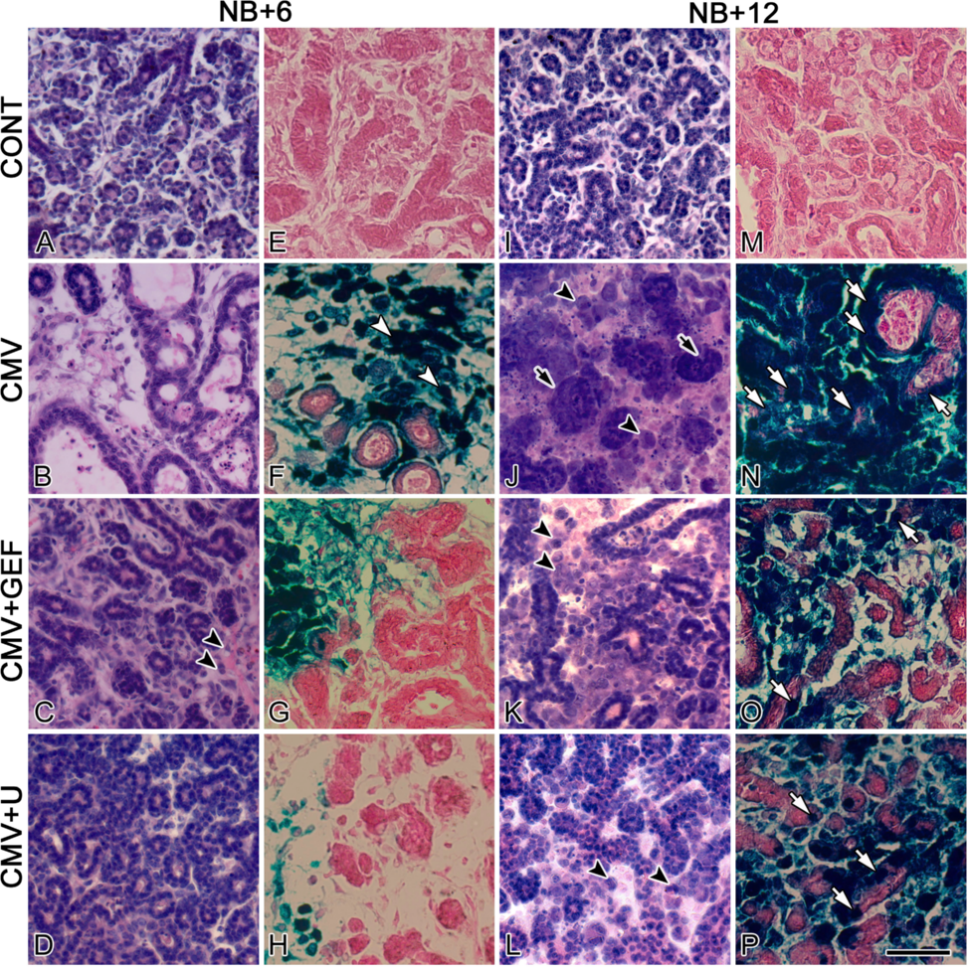
**Histology and mCMV distribution in NB+6 and NB+12 control, mCMV-infected, GEF-treated, mCMV-infected, and U0126-treated, mCMV-infected SMGs. A**-**H**. NB + 6 SMGs. **I**-**P**. NB + 12 SMGs. Control SMGs (**A**, **E**) exhibit normal ductal and pro-acinar epithelia and fibromyxoid stroma containing loosely-packed stellate to ovoid fibroblasts; note the absence of β-galactosidase staining (**E**, **M**). mCMV-infected NB + 6 SMGs (**B**, **F**) exhibit stromal viral CPE in stroma, decreased epithelia with dilated lumina, and β-galactosidase-stained virus in peripheral stromal. In GEF-treated, mCMV-infected NB + 6 SMGs (**C**, **G**), an improved phenotype consists of a fibromyxoid stroma, and decreased β-galactosidase-stained virus. However, reduced epithelial branching and occasional viral CPE (double black arrowheads) persists. In U0126-treated, mCMV-infected NB + 6 SMGs (**D**, **H**), the greatly improved phenotype approximates controls; these SMGs exhibit regularly branched epithelia, sparse fibromyxoid stroma, and no ductal dilation or viral CPE; little β-galactosidase- is seen (**H**). I-P. NB + 12 SMGs. mCMV-infected NB + 12 SMGs (**J**, **N**) are characterized by poorly-differentiated and dysplastic epithelia (arrows), abnormal stroma composed of sheets of small eosinophilic and larger basophilic, pleiomorphic cells, some exhibiting pathognomonic kidney-shaped nuclei (arrowheads). And densely-packedβ-galactosidase-stained virus throughout abnormal stroma and epithelia. GEF-treated, mCMV-infected SMGs (**K**, **O**) lost the improved phenotype seen on day 6 (compare **K** to **C**); they exhibit epithelial dysplasia, viral CPE in the stroma, pathognomonic kidney-shaped nuclei (black arrowheads), and β-galactosidase staining throughout abnormal stroma and sparsely in epithelia. U0126-treated, mCMV-infected SMGs (**L**, **P**) lost the improved phenotype seen on day 6, with overt evidence of epithelial dysplasia, viral CPE, pathognomonic kidney-shaped nuclei (black arrowheads), and β-galactosidase-staining throughout abnormal stroma and sparsely in epithelia. Reduced β-galactosidase staining is seen in GEF- and U0126-treated SMGs compared to untreated SMGs (compare O, P to N). Bar: **A-D, I-L** - 50 μm; **E**-**H**, **M**-**P**- 40 μm.

**Figure 3:**
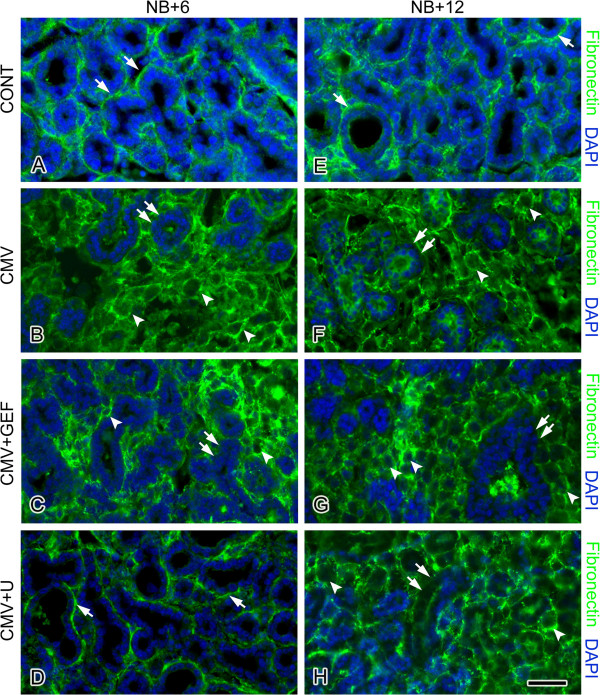
**Spatial distribution of FN on days 6 and 12 of culture. A**-**D**. NB + 6. **E**-**H**. NB + 12. Control NB + 6 (**A**) and NB + 12 (**E**) SMGs show a distinct and well-demarcated pattern of FN immunoreactivity at the BMZ (white arrows). In mCMV-infected SMGs, there is a marked change in FN localization in both NB + 6 (**B**) and NB + 12 (**F**) glands. FN immunostaining surrounds individual metaplastic stromal cells (white arrowheads), with a notable decline in FN immunoreactivity at the BMZ being seen (double white arrows). In GEF-treated, mCMV-infected NB + 6 SMGs (**C**), FN immunoreactivity is seen surrounding a small number of abnormal stromal cells (white arrowheads) and is mostly absent from BMZ (double white arrows). In U0126-treated, mCMV-infected SMGs (**D**), FN is immunolocalized at the BMZ (white arrow) in a pattern closely resembling that seen in controls (compare **D** to **A**) and markedly different from that seen in untreated, mCMV-infected SMGs (compare **D** to **B**). In contrast, the distribution of FN immunolocalization in GEF-treated (**G**) and U0126-treated (**H**), mCMV-infected NB + 12 SMGs resembles that seen in untreated mCMV-infected SMGs (compare **G**, **H** to **F**); FN immunoreactivity shows a pericellular distribution on stromal cells (white arrowheads) and near absence at the BMZ (double white arrows). Bar: **A**-**B**, **D**-**H**- 37 μm, **C**- 45 μm.

Newborn (NB) mouse SMG organs were cultured with 1 × 10^5^ PFU/ml mCMV for 24 h and maintained for 6 or 12 days; controls consisted of NB SMG organs cultured for identical periods in control medium. Control SMGs (Figures 1A, 2A, I) demonstrate densely packed, branched cuboidal epithelial cells within a sparse fibromyxoid stroma containing numerous stellate to ovoid fibroblasts. The epithelia is composed of both serous and mucous acini with associated ducts. Individual epithelial cells have uniformly sized, centrally-located, basophilic nuclei surrounded by eosinophilic cytoplasm. Regularly distributed, small-diameter, centrally-located ductal lumina are evident, often with pale staining mucous. As expected, fibronectin (FN) is clearly evident in the basement membrane zone (BMZ) of epithelial ducts and acini (Figures 1F, 3A, E).

**Figure 4:**
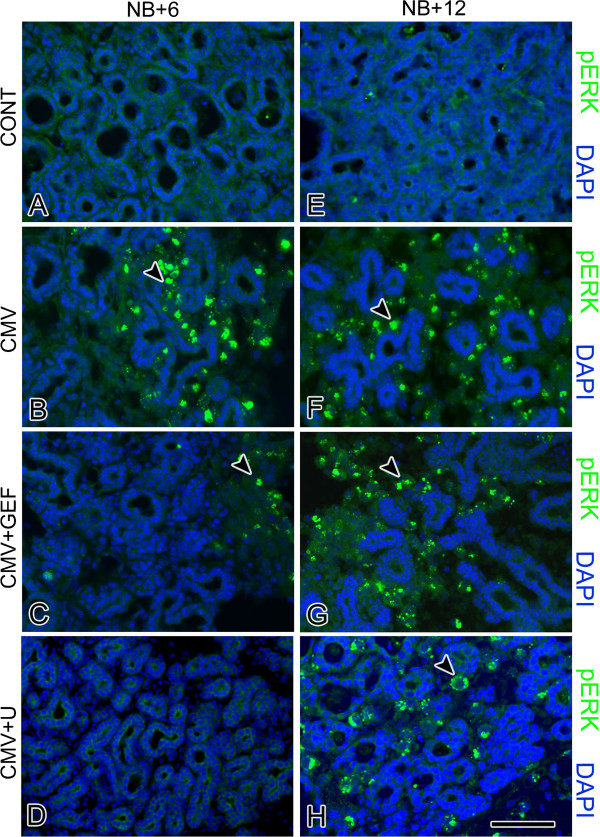
**Effect of EGFR/ERK inhibitors on the distribution of activated ERK (pERK) in mCMV-infected NB + 6 and NB + 12 SMGs. A-D**. NB + 6 SMGs. **E-H**. NB + 12 SMGs. mCMV induces a notable increase in pERK expression in NB + 6 (**B**) and NB + 12 (**F**) SMGs compared to uninfected NB + 6 (**A**) and NB + 12 (**E**) controls (compare **B** to **A** and **F** to **E**). GEF-treated (**C**) and U0126-treated (**D**) mCMV-infected NB + 6 SMGs show a notable decrease in immunodetectable pERK compared to untreated mCMV-infected SMGs (compare **C**, **D** to **B**); the near absence of pERK with U0126 treatment resembles that seen in controls (compare **D** to **A**). By day 12, there is a marked increase in pERK expression in both GEF-treated (**G**) and U0126-treated (**H**) mCMV-infected SMGs compared to day 6 (compare **G** to **C** and **H** to **D**); pERK expression more closely resemblesuntreated, mCMV-infected SMGs seen on day 6 (compare **G**, **H** to **B**) than to controls (compare **G**, **H** to **E**). Bar: A-F, H- 50 μm, G- 60 μm.

mCMV-infected NB + 6 and NB + 12 SMGs are characterized by viral cytopathic effect (CPE) in the stroma and abnormal parenchyma, altogether consistent with a tumorigenic phenotype (Figures 1B-E, 2B, J). There is a notable increase in mCMV infection between day 6 and 12 of culture (compare Figure 2N to 2F). On day 6, mCMV initially infects peripherally-localized stromal cells (Figure 2F); the absence of β-galactosidase-stained virus in epithelia suggests that epithelial pathogenesis is mediated by stromal-derived paracrine factors. By day 12, β-galactosidase-stained virus is densely distributed throughout abnormal stroma, and in epithelial ductal cells (Figure 2N). In mCMV-infected NB + 12 SMGs, there is a marked increase in stromal cellularity and abnormal epithelia compared to controls (compare Figures 1B-E to A, 2J to I). The abnormal stroma is composed of sheets of small eosinophilic cells and larger basophilic, pleiomorphic cells. Individual stromal cells are characterized by high nuclear-to-cytoplasm ratios, prominent nuclei and nucleoli, and frequent kidney-shaped nuclei pathognomonic of viral infection. In addition, there is a dramatic decline in epithelial ductal and acinar branching compared to controls; abnormal ducts and acini are composed of hyperplastic or pseudostratified epithelia and frequently exhibit severely dilated lumina. Individual epithelial cells exhibit increased nuclear-to-cytoplasm ratios, hyperchromatism, and visible nucleoli. Importantly, epithelial cells exhibit a spectrum of morphotypes, from dysplastic to *in situ* to invasive. Intra-luminal and extra-basal proliferation and migration of epithelial cells, lack of a distinct BMZ, and reduced cell-cell junctions impart a multi-layered appearance to many of the epithelial islands. Pyknosis, karyorrhexis and karyolytic debris within lumina are also associated with some of the epithelial islands. Kidney-shaped nuclei pathognomonic of CMV infection are frequently seen in stromal and ductal epithelial cells (Figure 1C-E). Distinct from controls, FN surrounds individual metaplastic stromal cells, and is relatively absent from the BMZ of branched epithelia (compare Figures 1G, H to F, 3B, F to 3A, E). Additionally, immunodetectable pERK is seen in abundance (Figure 4B, F), being absent in controls (Figure 4A, E).

**Figure 5:**
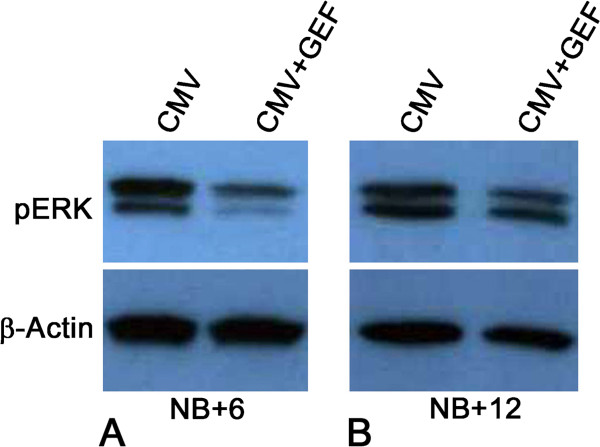
**Western blot analysis of pERK protein expression in mCMV-infected and GEF-treated, mCMV-infected NB + 6 and NB + 12 SMGs. A**. NB + 6 SMGs. **B**. NB + 12 SMGs. There is a significant increase in pERK expression in GEF-treated, mCMV-infected NB + 12 SMGs compared to identically treated NB + 6 SMGs (p<0.01; n = 16). GEF treatment results in a significant decrease in pERK expression in mCMV-infected NB + 6 SMGs compared to untreated SMGs (p<0.01; n = 9), but no difference is seen between mCMV-infected and GEF-treated, mCMV-infected NB + 12 SMGs (p>0.4; n = 9).

Of particular concern here is the emerging resistance to EGFR→ ERK pathway inhibitors (Figures 2, 3 and 4). Gefitinib (GEF) blocks the binding of ATP to the intracellular tyrosine kinase domain of EGFR and thus inhibits downstream ERK 1/2 activation and cell proliferation, as well as promotes cell cycle arrest at the G_1_–S boundary and apoptosis [11,12]. In these studies, NB SMGs were infected with 1 × 10^5^ PFU/ml mCMV for 24 hrs in the presence or absence of 10 μM GEF and then cultured in control medium with or without GEF for a total of 6 or 12 days. As previously reported [5], after 6 days in culture (NB + 6), mCMV-infected, GEF-treated SMGs present a much improved phenotype (Figures 2C, 3C). There is a marked increase in normal epithelial branching, and no ductal dilation. FN immunostaining is seen in a subset of epithelial BMZ and on stromal cells; FN is also absent from a subset of epithelial BMZs (Figure 3C). There is predominantly a fibromyxoid stroma containing stellate fibroblasts, and only occasional viral CPE. This “near rescue” of the mCMV-induced pathology of NB + 6 SMGs with GEF inhibition of EGFR phosphorylation is coincident with reduced mCMV infection compared to un-treated SMGs (compare Figure 2G to F), as well as a highly significant (p<0.01) decline in downstream pERK protein expression (compare Figure 4C to B; Figure 5A).

After 12 days in culture (NB + 12), mCMV-infected, GEF-treated SMGs present a regressive phenotype with overt evidence of epithelia dysplasia, viral CPE in the stroma (Figures 2J, 3G, 4G), and a notable increase in mCMV infection throughout stroma compared to day 6 (compare Figure 2O to G). Epithelial branching is markedly reduced, ductal lumina are frequently dilated, and individual epithelial cells have increased nuclear-to-cytoplasmic ratios, hyperchromatism, and visible nucleoli. The stroma contains sheets of small eosinophilic and large, basophilic, pleiomorphic cells with high nuclear-to-cytoplasmic ratios, prominent nuclei and nucleoli and frequent pathognomonic kidney-shaped nuclei. Again, FN surrounds individual metaplastic stromal cells and is relatively absent from the BMZ of epithelia (Figure 3G). By day 12, there is a marked increase in pERK protein expression in GEF-treated, mCMV-infected SMGs relative to day 6 (p<0.01); by day 12, there is no significant difference (p>0.4) between GEF-treated and non-treated mCMV-infected SMGs (compare Figure 4G to C, G to F; Figure 5).

We had previously speculated that ERK activation by signaling pathways other than EGFR might explain the incomplete GEF-mediated rescue of mCMV-induced pathology in NB + 6 SMG organs [5]. We tested this hypothesis by direct inhibition of ERK activation using U0126, a small molecule inhibitor of MEK-mediated ERK phosphorylation (e.g. [13]). In these experiments, NB SMGs were infected with 1 × 10^5^ PFU/ml mCMV for 24 hrs in the presence or absence of 10 μM U0126 and then cultured in control medium with or without U0126 for a total of 6 or 12 days. As previously reported [5], U0126-treated, mCMV-infected NB + 6 SMGs are indistinguishable from controls (compare Figure 2D to A): normal branching epithelia, no ductal dilation, sparse fibromyxoid stroma, FN immunolocalized to epithelial BMZ, and no evidence of pERK protein expression (Figures 3D, 4D). This rescue is likely due to the near absence of mCMV infection (Figure 2H). Nevertheless, by NB + 12, mCMV-infected, U0126-treated SMGs present a metaplastic phenotype not unlike that seen with GEF treatment on day 12, even if less severe, including a marked upregulation of pERK protein expression compared to day 6 (Figures 2L, 3H, 4H). mCMV burden is similar to that seen in GEF-treated NB + 12 SMGs (compare Figure 2P to O) and notably more than on day 6 (compare Figure 2P to H).

### Co-targeted inhibition of the EGF → ERK pathway

Tyrosine autophosphorylation of the intracellular domain of EGFR results in the recruitment of the GRB2/SOS signaling complex, GTP-loading of the proximate Ras, and subsequent activation of Raf kinase and a phosphorylation cascade from MEK to ERK. Importantly, the ERK pathway resembles a negative feedback amplifier (NFA) with the amplifier consisting of the three-tiered kinase module Raf-MEK-ERK and negative feedbacks emanating from ERK to SOS and Raf; the ratio of protein abundances of Raf, MEK and ERK is about 1:3:6 [13]. This NFA is much like similar design principles used in electronic circuits to confer robustness, output stabilization, and linearization of nonlinear signal amp-lification [13]. These properties are determinative of both activation kinetics and response to small molecule inhibitors. Having already established that there is fairly rapid resistance to individual inhibitors of nodes outside (EGFR/gefitinib) and inside (MEK/U0126) the NFA (Figures 2, 3, 4 and 5), we needed to delineate an alternative strategy to break the NFA. To preclude resistance in the long-term, modeling and cell culture studies have suggested *concurrently* inhibiting targets outside and inside the NFA [13,14].

**Figure 6:**
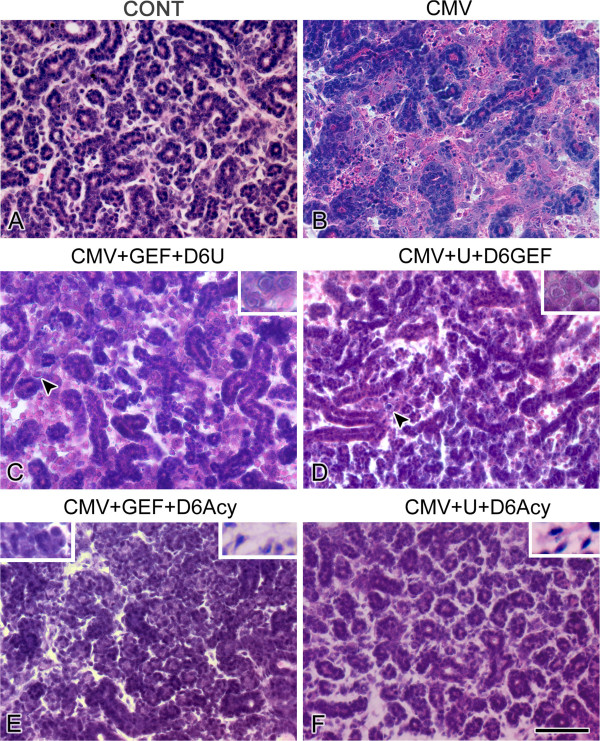
**Effect of co-targeting pathways on histopathology in mCMV-infected NB + 12 SMGs. A**. Control SMG. **B**-**F**. mCMV-infected SMGs. mCMV-infected SMGs (**B**) show pathology composed of abnormal epithelia and viral CPE in the stroma. GEF-treated, mCMV-infected SMGs treated with U0126 beginning on day 6 (CMV + GEF + D6U) (**C**) exhibit progressive pathology but continues to present decreased branching and stromal pathology with viral CPE (arrowhead) (compare **C** to **B**). Inset: Higher magnification of mCMV-infected and affected stromal cells. U0126-treated, mCMV-infected SMGs treated with GEF beginning on day 6 (CMV + U + D6 GEF) (**D**) exhibit progressive pathology and demonstrates improved epithelial morphology and branching (compare **D** to **B**, **C**), with continued viral CPE in stroma (arrowhead) being seen (compare **D** to **A**). Inset: Higher magnification of mCMV-infected and affected stromal cells. GEF-treated, mCMV-infected SMG (**E**) treated with aciclovir beginning on day 6 (CMV + GEF + D6 Acy) demonstrate a near normal morphology of extensive epithelial branching, abundant serous acini, and a fibromyxoid stroma (compare **E** to **A**). Right Inset: Higher magnification of stromal stellate fibroblasts. Left Inset: Higher magnification of serous acini. U0126-treated, mCMV-infected SMG (**F**) treated with aciclovir beginning on day 6 (CMV + U + D6 Acy) demonstrate a near normal morphology, composed of a highly branched epithelia in a fibromyxoid stroma approximating control SMGs (compare **F** to **A**). Inset: Higher magnification of stromal stellate fibroblasts. Bar: **A**-**F**, 50 μm; Insets **C**, **D**, **F** - 25 μm. **E**, left inset- 30 μm; right inset - 25 μm.

**Figure 7:**
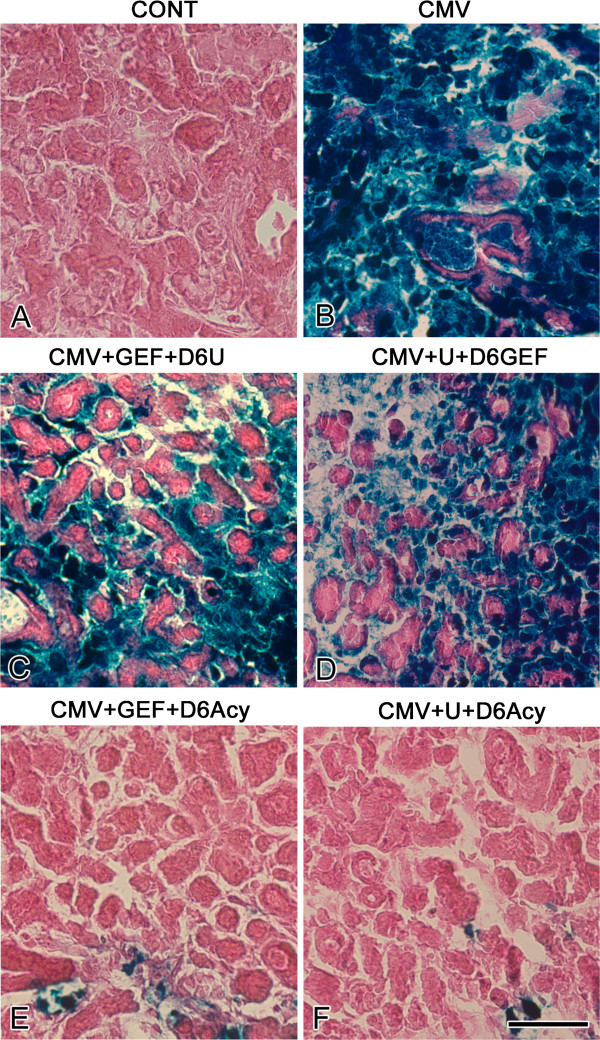
**The effects of co-targeting pathways on viral distribution in NB + 12 SMGs.** In control SMGs (**A**), uninfected epithelia and stroma appear pink. In mCMV-infected SMGs (**B**), densely-packed β-galactosidase-stained virus is seen thoughout stroma and in epithelial duct cells. In GEF-treated, mCMV-infected SMGs treated with U0126 beginning on day 6 (**C**), there is a reduction in β-galactosidase-stained virus in stroma and a more notable reduction in epithelia compared to untreated SMGs (compare **C** to **B)**. In U0126-treated, mCMV-infected SMGs treated with GEF beginning on day 6 (**D**), there is a greater reduction in β-galactosidase-stained virus than seen in SMGs initially treated with GEF (compare **D** to **C**). Addition of aciclovir beginning on day 6 to GEF-treated, mCMV-infected SMGs (**E**) and U0126-treated, mCMV-infected SMGs (**F**) results in the near absense of β-galactosidase-stained virus. Bar: **A**-**C**, **E**-**F**, 50 μm; **D** 60 μm.

**Figure 8:**
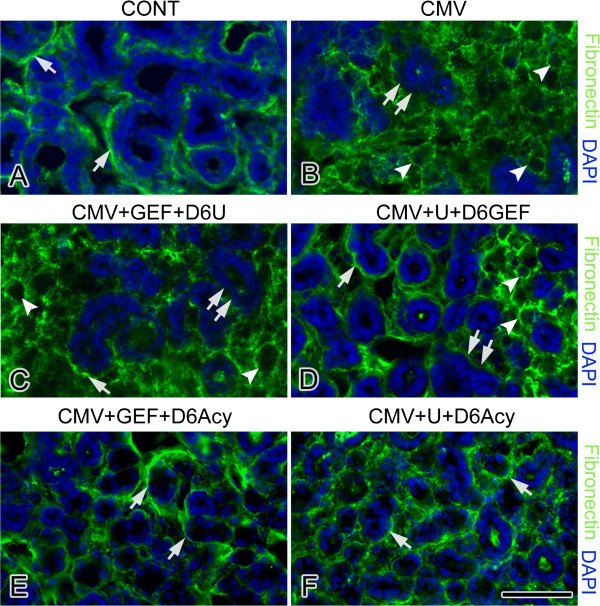
**The effects of co-targeting pathways on FN distribution in NB + 12 SMGs.** Control SMGs (**A**) show a distinct and well-demarcated pattern of FN immunoreactivity at the BMZ (white arrow). B-F. mCMV-infected SMGs. In mCMV-infected SMGs (**B**), FN immunoreactivity is found surrounding abnormal stromal cells (white arrowheads) but is relatively absent at the epithelial BMZ (double white arrow). In GEF-treated, mCMV-infected SMGs treated with U0126 beginning on day 6 (**C**), FN immunostaining is primarily seen surrounding stromal cells (white arrowhead) and is mostly absent from BMZ (double white arrows); FN is sporadically seen in BMZ (white arrow). In U0126-treated, mCMV-infected SMGs treated with GEF beginning on day 6 (**D**), FN is immunolocalized both at the BMZ (white arrow) and surrounding abnormal stromal cells (white arrowheads) but is absent from the BMZ of a subset of epithelial islands (double white arrows). The addition of aciclovir treatment beginning day 6 to GEF-treated, mCMV-infected SMGs (**E**) or U0126-treated, mCMV-infected SMGs (**F**) results in a more normal staining pattern; FN immunostain is primarily seen at the BMZ (white arrow) in a pattern resembling that seen in controls (compare **E**, **F** to **A**). Bar: 38 μm.

**Figure 9:**
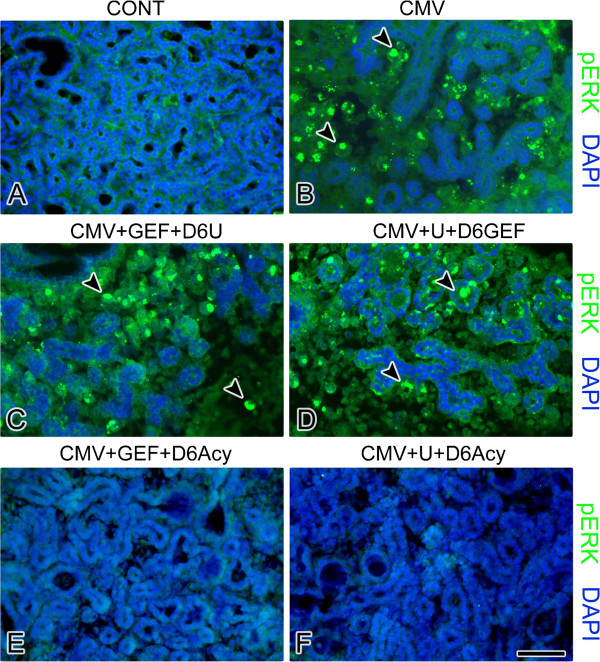
**The effects of co-targeting pathways on the spatial expression of pERK in NB + 12 SMGs.** In control SMGs (**A**), pERK immunostaining is mostly absent. mCMV-infected SMGs (**B**) are characterized by a marked increase in pERK expression in stromal cells (arrowheads) compared to control (compare **B** to **A**). In GEF-treated, mCMV-infected SMGs treated with U0126 beginning on day 6 (**C**) or U0126-treated, mCMV-infected SMGs treated with GEF beginning on day 6 (**D**), pERK immunoreactivity is seen in stromal cells (arrowheads) (compare **C**, **D** to **A**). The addition of aciclovir treatment beginning on day 6 to GEF-treated SMGs (**E**) or U0126-treated, mCMV-infected SMGs (**F**) results in the near absence of pERK immunoreactivity which closely resembles controls (compare **E**, **F** to **A**). Bar: A-B, E-F- 50 μm; **C**-**D**- 55 μm.

In these studies, NB SMGs are infected with 1 × 10^5^ PFU/ml mCMV for 24 hr in the presence of either 10 μM GEF or 10 μM U0126 and then cultured to day 12 in control medium with the respective inhibitor along with the addition of either 10 μM U1026 (GEF + D6U) or 10 μM GEF beginning on day 6 (U + D6GEF). GEF-treated, mCMV-infected SMGs co-treated beginning on day 6 with U0126 demonstrate morphologic improvement without ductal dilation (compare Figure 6C to B), but there is continued evidence of decreased epithelial branching and stromal pathology with viral CPE (compare Figure 6C to A). This improvement is coincident with reduced mCMV burden compared to untreated SMGs (compare Figure 7C to B). FN is still primarily seen surrounding stromal cells (Figure 8C); pERK continues to be expressed at high levels (Figure 9C). For U0126-treated, mCMV-infected SMGs co-treated beginning on day 6 with GEF, the morphological phenotype and viral load are similar to that seen in GEF + D6 U0126 treated NB + 12 SMGs (compare Figure 6D to C, Figure 7D to C). Interesting differences include increased epithelial branching (compare Figure 6D to C) with the reappearance of FN at the BMZ of acini and ducts along with surrounding abnormal stromal cells (Figure 8D), and a marginally diminished pERK protein expression (Figure 9D). Thus, though the rescue is incomplete, co-targeted inhibition of the EGFR →ERK pathway precludes the regression to the more severe pathology seen with single-targeted inhibition (compare 6C to 2k and 6D to 2L).

### mCMV-dependence and molecular correlates

The extensive effort to identify resistance mechanisms has uncovered a recurrent theme, namely the utilization of survival signals redundant to those transduced by the targeted kinase or kinase pathway [15,16]. CMV has evolved numerous strategies for dysregulating host cell signaling in order to propagate viral progeny [6,17]. Further, we have previously shown that when SMGs *in vitro* are infected with mCMV for 24 h and then cultured for 6–12 days in the presence of the antiherpesviral nucleoside, aciclovir, mCMV replication is suppressed and the SMGs are histologically and molecularly normal [4-6]. Thus, we tested the hypothesis that addition of aciclovir beginning on day 6, along with the EGFR/ERK pathway inhibitor, would preclude progressive pathway dysregulation and rescue the infected NB SMGs.

In these experiments, NB SMGs were infected with 1 × 10^5^ PFU/ml mCMV for 24 h in the presence of either 10 μM GEF or 10 μM U0126 and then cultured to day 12 in control medium with the respective inhibitor along with the addition of 10 μg/ml aciclovir beginning on day 6 (CMV + GEF + D6Acy; CMV + U0126 + D6 Acy). GEF-treated, mCMV-infected SMGs treated with aciclovir beginning on day 6 show normal epithelial branching and normal fibromyxoid stroma (Figure 6E), as well as the near absence of β-galactosidase-stained virus (Figure 7E). The distribution patterns of FN and pERK are similar to controls; FN is localized to the BMZ of epithelia and there is no detectable pERK protein expression (compare Figures 8E to A, 9E to A). The results of U0126-treated SMGs with aciclovir beginning on day 6 are identical (Figures 6F, 7F, 8F, 9F). Clearly the maintenance of SMG pathology is dependent upon (“addicted to,” if you will) active and robust viral replication (Figures 2M-P, 7), expression of late viral gene products, and the concomitant subversion of multiple host cell signaling pathways.

Gene expression profiles of a limited number (n = 6) of human SG MEC specimens have revealed a significant upregulation of FN (FN) and interleukin 6 (IL-6) [18]. We have previously found that mCMV induced pathogenesis in embryonic mouse SMGs is characterized by the upregulation of several major ERK 1/2 related pathways in addition to EGFR, including FN and IL-6 [6]. Here we examined both FN and IL-6 protein cell-specific distribution relative to the mCMV-induced pathologic phenotype in NB SMGs.

We have shown above that mCMV infection of NB SMGs dysregulates FN protein expression on days 6 and 12 of culture, namely upregulation and cytologic redistribution (Figures 3A, B, E, F; 8A, B). This is somewhat ameliorated by day 6 with GEF treatment (Figure 3C) and eliminated with U0126 treatment (Figure 3D). However, only the addition of the antiviral, aciclovir, beginning on day 6 of culture, precludes progressive FN dysregulation, and effects concomitant rescue of infected NB SMGs by day 12 (compare Figures 8E, F to 3G, H).

**Figure 10:**
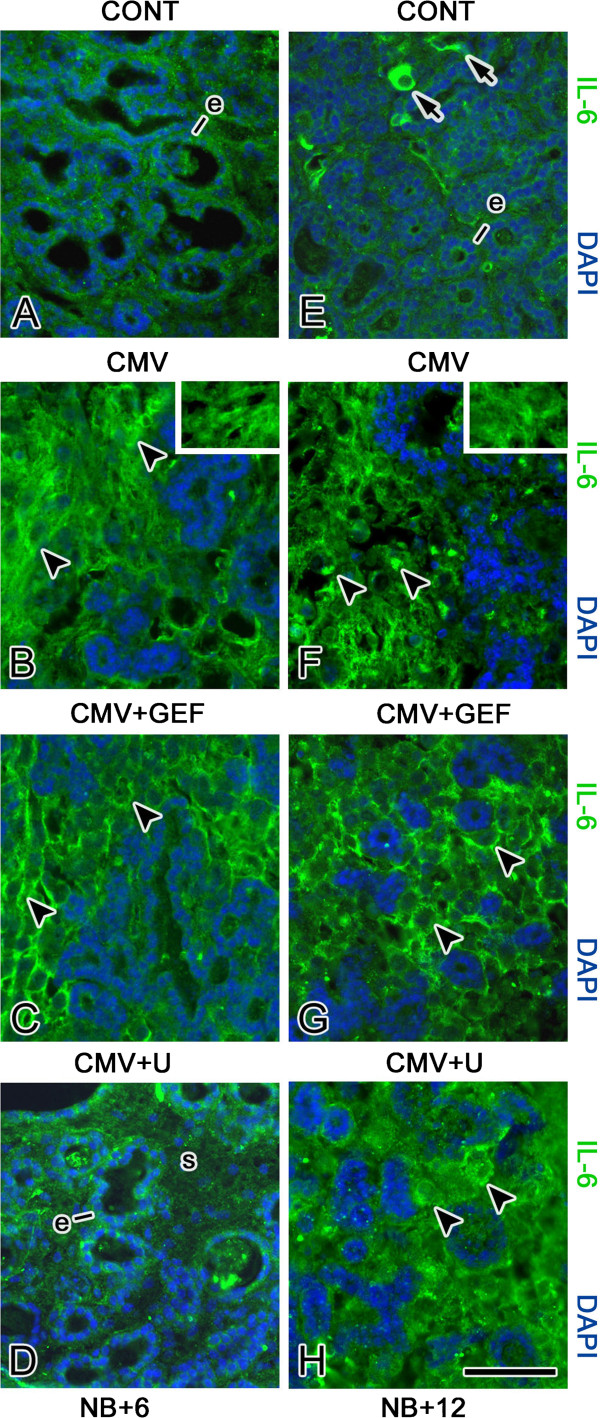
**mCMV-induced changes in the spatial distribution of IL-6 on days 6 and 12 of culture. A-D**. NB + 6 SMGs. **E-H**. NB + 12 SMGs. In controls (**A**, **E**), IL-6 immunostain is weakly seen on ductal and proacinar epithelia (e); it is also localized in nerves (arrows). With mCMV infection (**B**, **F**), there is a marked increase in immunodetectable IL-6 in abnormal stromal cells (arrowheads) but is relative absent from epithelia. Insets: Higher magnification of IL-6 stained stromal cells. In GEF-treated, mCMV-infected NB + 6 SMGs (**C**), IL-6 immunoreactivity is seen primarily on stromal cells (arrowheads). In U0126-treated, mCMV-infected NB + 6 SMGs (**D**), IL-6 immunostaining resembles that seen in controls; IL-6 is weakly seen on epithelia (e) but absent from stroma (s). On day 12, GEF-treated, mCMV-infected (**G**) or U0126, mCMV-infected (**H**) SMGs exhibit IL-6 immunoreactivity throughout the stromal cells (black arrowheads) and absence from epithelia. Bar: 50μm; Insets **B**, **F** -30 μm.

**Figure 11:**
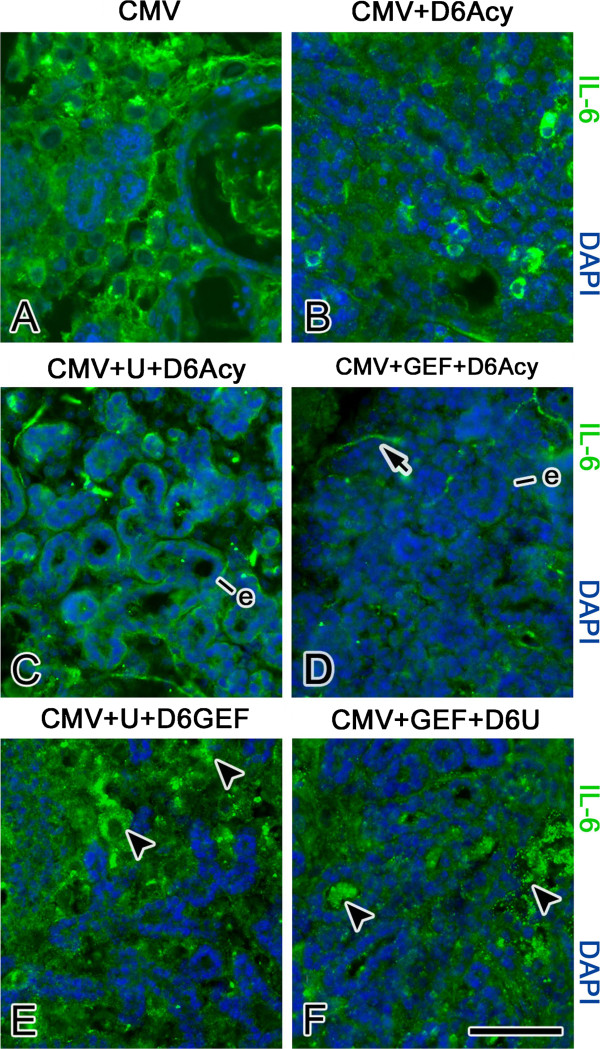
**The effects of antivirals with/without co-targeting the EGFR/ERK pathway on IL-6 protein expression in NB + 12 SMGs.** mCMV infection (**A**) induces a notable increase in immunodetectable IL-6 in abnormal stromal cells. The addition of aciclovir beginning on day 6 (CMV + D6 Acy) (**B**) substantially reduces IL-6 immunostaining in stroma. When aciclovir was added beginning on day 6 to U0126-treated (CMV + U + D6 Acy) (**C**) or GEF-treated (CMV + GEF + D6Acy) (**D**) SMGs, the distribution of IL-6 immunostaining resembles control (compare **C**, **D** to 10 **A**, **E**), being detected in epithelia (e) and nerve cells (arrow) and absent from stroma. In contrast, IL-6 immunolocalization persists in stromal cells (arrowheads) in both U0126-treated SMGs treated with GEF beginning on day 6 (CMV + U + D6GEF) (**E**) or GEF-treated SMGs treated with U0126 beginning on day 6 (CMV+ GEF+ D6U) (**F**). Bar: **A**, **B**, **F**- 40 μm; **C**-**E** - 50 μm.

With respect to IL-6, mCMV infection of NB SMGs through day 12 of culture is associated with both overexpression and redistribution of IL-6 protein from epithelia to abnormal stromal cells (compare Figure 10B, F to A, E). In GEF-treated SMGs, the pathology seen on day 6 of culture is concomitant with overexpression and localization of IL-6 to the abnormal stromal cells (Figure 10C). In contrast, U0126-treated, mCMV-infected NB + 6 SMGs exhibit a localization pattern similar to that seen in controls (compare Figure 10D to A). In all treatment groups (GEF; U0126; GEF + D6U0126; U0126 + D6GEF), the pathology seen on day 12 of culture is concomitant with overexpression and localization of IL-6 to the abnormal stromal cells, with a slightly improved localization pattern being seen with pathway co-targeting (Figures 10G-H, 11E-F). Only the addition of aciclovir beginning on day 6 to either GEF (GEF + D6Acy) or U0126 (U + D6Acy) precludes progressive IL-6 dysregulation, and effects concomitant rescue of infected NB SMGs by day 12 of culture (Figure 11C, D). Aciclovir treatment alone results in a somewhat improved IL-6 distribution pattern, with IL-6 being seen on fewer stromal cells than in untreated, mCMV-infected SMGs (compare Figure 11B to A).

## Discussion

As one element of a certain multifactorial etiology, we recently identified a virally implicated relationship between active human CMV and mucoepidermoid carcinoma of the human salivary glands [3]. More than 90% of MEC specimens uniformly correlate with active hCMV protein expression and an upregulation and activation of the EGFR →ERK pathway. Even though targeting this pathway would appear to be a good therapeutic approach, human trials with small molecule protein kinase inhibitors (PKIs) have met with limited initial success, increasing drug resistance and subsequent progressive tumorigenesis [15,19,20].

Using a mouse 3D organ culture model of CMV-induced cellular pathology which resembles secretory glandular neoplasia [4,5], we have identified several emergent phenomena that are likely dispositive clues to the mechanism of resistance: (1) while there is short term rescue, mouse SMGs tumors soon reveal an acquired resistance to EGFR → ERK pathway protein kinase inhibitors (PKIs), alone or in combination; (2) SMG neoplasia is dependent upon the continued activity of CMV (“viral addiction”) and, thus, long term tumor regression can only be sustained by concurrent PKI and antiviral treatment; (3) CMV-dependent, PKI resistance is associated with ectopic overexpression of FN and IL-6 proteins in abnormal stromal cells. These observations may have important therapeutic implications for human salivary gland tumors.

Two key resistance mechanisms appear to be increased expression/activation of the targeted kinase, and use of alternative signaling to activate downstream cell survival pathways [15]. More specifically, tumor cells typically upregulate multiple pathways that mediate signals which share common critical downstream effectors, particularly PI3K/AKT and MEK/ERK transduction [16]. IL-6/IL-6R and FN/integrin binding initiate multifunctional signaling (PI3K/AKT and MEK/ERK) that mediates cell growth, differentiation and survival in development and progressive tumorigenesis [21-25]. Further, there is mutual cross-talk between FN/integrin and EGFR [26]. It would appear, then, that CMV-dependent FN and IL-6 overexpression in abnormal stromal cells increases the activation of the targeted kinase, ERK (Figures 3, 4, 8, 9, 11) and likely induces alternative signaling (e.g. PI3K/AKT; FN/INT/EGFR). Thus, in the presence of active CMV (Figures 2O, P, 7C, D), even the highest nontoxic dose of MEK → ERK inhibitor (U0126) is unable to preclude progressive tumorigenesis (Figure 2L), nor is the highest dose of EGFR inhibitor (GEF) (Figure 2K) or combination of inhibitors (Figure 6C, D).

These results permit a glimpse of the complexity before us. Given the considerable pathway crosstalk and redundancy in mammalian cells and the multifunctional paths mediated by single molecular components, elucidating the precise effect of a virus on the host “interactome” is quite daunting [27]. Nevertheless, systematic analyses of host targets can identify dysregulated host cell networks and potentially reveal all pathways that go awry in virally implicated tumorigenesis [28]. Ultimately, it is a problem amenable to quantitative systems analysis, not unlike those in embryonic development and differentiation [8].

## Conclusions

We report that although EGFR → ERK pathway inhibition initially attenuates tumor progression and induces tumor regression, it is uniformly limited by an acquired drug resistance, and subsequent failure to sustain either tumor regression or stability. Long term tumor regression can only be sustained by concurrent kinase inhibitor and antiviral treatment. The resistance to kinase inhibitors is dependent upon CMV dysregulation of alternative pathways with downstream effectors common with the targeted pathway, a phenomenon with important therapeutic implications for human MEC of salivary glands.

## Abbreviations

AREG: Amphiregulin; BMZ: Epithelial basement membrane zone; CMV: Cytomegalovirus; CPE: Cytopathic effect; CRTC1: CREB-regulated transcription coactivator 1; D6U: Addition on U0126 beginning on day 6; D6GEF: Addition of gefitinib beginning on day 6; D6 ACY: Addition of aciclovir beginning on day 6; ERK1/2: Extracellular signal-related kinase 1/2; FN: Fibronectin; GEF: Gefitinib; hCMV: Human CMV; IL-6: Interleukin 6; mCMV: Mouse CMV; MEC: Mucoepidermoid carcinoma; MEK: Mitogen-activated ERK kinase; NB: Newborn; NFA: Negative feedback amplifier; pERK: Phosphorylated ERK1/2; PFU: Plaque forming units; PKI: Protein kinase inhibitor; SG: Salivary glands; SMG: Submandibular glands

## Competing interests

The authors declare that there are no conflicts of interests.

## Authors’ contributions

TJ and MM conceived and designed the study. MM coordinated all experiments and drafted the manuscript. TJ was involved in all experiments and analyses, generated figures, and helped draft the manuscript. PS participated in the analysis of pathology and helped draft the manuscript. KD was involved in conducting experiments, data collection and generating figures. All authors have read and approved the final manuscript.
